# Voxel Size and Field of View Influence on Periodontal Bone Assessment Using Four CBCT Systems: An Experimental Ex Vivo Analysis

**DOI:** 10.3390/tomography11070074

**Published:** 2025-06-25

**Authors:** Victória Geisa Brito de Oliveira, Polyane Mazucatto Queiroz, Alessandra Rocha Simões, Mônica Ghislaine Oliveira Alves, Maria Aparecida Neves Jardini, André Luiz Ferreira Costa, Sérgio Lucio Pereira de Castro Lopes

**Affiliations:** 1 Department of Diagnosis and Surgery, The Institute of Sciences and Technology of São Paulo State University (UNESP), São José dos Campos 12247-014, SP, Brazil; victoria.gb.oliveira@unesp.br (V.G.B.d.O.); maria.jardini@unesp.br (M.A.N.J.); sergio.lopes@unesp.br (S.L.P.d.C.L.); 2 Department of Dentistry, Ingá University Center, Maringa 87035-510, PR, Brazil; polyanequeiroz@hotmail.com; 3 HD Oral Radiology Center, São João-Del-Rei 36305-170, MG, Brazil; alessandrasimoes@hotmail.com; 4 Department of Biosciences and Oral Diagnosis, The Institute of Sciences and Technology of São Paulo State University (UNESP), São José dos Campos 12247-014, SP, Brazil; monica.alves@unesp.br; 5 Postgraduate Program in Dentistry, Cruzeiro do Sul University (UNICSUL), São Paulo 01506-000, SP, Brazil

**Keywords:** alveolar bone loss, cone-beam computed tomography, field of view, measurement accuracy, periodontal diagnosis, voxel size

## Abstract

Objective: This ex vivo study aimed to evaluate the influence of different acquisition protocols, combining voxel size and field of view (FOV), across four cone-beam computed tomography (CBCT) systems, on the accuracy of alveolar bone level measurements for periodontal assessment. Materials and Methods: A dry human mandible was used, with standardized radiopaque markers placed on the cementoenamel junction (CEJ) of the buccal–mesial and buccal–distal aspects of teeth 34 and 43. CBCT scans were performed using four systems—Veraview^®^ X800, OP300 Pro^®^, I-CAT Next Generation^®^, and Orthophos XG^®^—applying various combinations of field of view (FOV) and voxel resolution available in each device. Reference measurements were obtained in situ using a digital caliper. CBCT images were exported in DICOM format and analyzed with OnDemand3D software (version 4.6) to obtain paracoronal sections. Linear measurements from the CEJ to the alveolar crest were recorded in triplicate and compared to the gold standard using ANOVA and the Dunnett test (α = 0.05). Results: Protocols with smaller voxel sizes and limited FOVs generally yielded measurements closer to the gold standard. However, some larger-FOV protocols with intermediate voxel sizes also achieved comparable accuracy. Among the systems, the I-CAT showed lower agreement within in situ measurements, while others demonstrated reliable performance depending on the acquisition parameters. Conclusions: The findings suggest that CBCT protocols with smaller voxel sizes and reduced FOVs can enhance measurement accuracy in periodontal bone assessments. Nevertheless, intermediate protocols may offer a balance between diagnostic quality and radiation exposure, aligning with the ALADA principle. This study reinforces the need for standardized acquisition parameters tailored to periodontal imaging.

## 1. Introduction

Periodontitis is a multifactorial, chronic inflammatory disease associated with dysbiotic biofilm, resulting in the progressive destruction of periodontal attachment structures. Diagnosis is primarily established through clinical probing of periodontal pockets, complemented by radiographic imaging, using the cementoenamel junction (CEJ) as the anatomical reference for bone level assessment. Alveolar bone loss is considered both a key indicator and a consequence of periodontal disease progression [1].

Panoramic and intraoral radiographs are typically the first-line imaging modalities for periodontal evaluation. However, their inherent limitation lies in their two-dimensional (2D) nature, which can lead to under- or overestimation of bone levels and hinder the characterization of bony defects. In contrast, cone-beam computed tomography (CBCT) offers three-dimensional (3D) imaging with superior anatomical detail, allowing more accurate and noninvasive visualization of osseous structures, which is of significant value in clinical periodontal practice. Current evidence supports the use of CBCT for evaluating alveolar crest height, the presence and extent of periodontal bone defects, and pre- and postoperative assessment in periodontal surgery [2,3,4,5,6,7].

Several technical parameters influence CBCT image quality, notably voxel size—which determines spatial resolution—and the field of view (FOV), which defines the scanned anatomical volume. Larger voxel sizes reduce spatial resolution, whereas larger FOVs increase the amount of irradiated tissue and may incorporate unnecessary structures. Both factors directly affect radiation dose and diagnostic efficacy [8]. Although CBCT is increasingly used in dentistry, there is still no consensus or standardized acquisition protocol specifically tailored for periodontal assessment. Furthermore, clinical decision-making should adhere to the ALADA principle (As Low As Diagnostically Acceptable), which emphasizes achieving diagnostic sufficiency while minimizing radiation exposure [9].

As reported in the literature, voxel size is directly associated with image resolution: an increase in voxel size results in decreased spatial resolution and, consequently, reduced detail quality. Regarding the field of view (FOV), smaller FOVs tend to reduce image scatter and artifacts while also lowering the patient’s radiation exposure [10]. Technically, protocols using smaller voxel sizes and FOVs can improve image quality; however, they may also increase radiation dose due to higher resolution demands. Dillenseger et al. [11] evaluated only FOV as a variable and observed that images acquired with smaller FOVs yielded diagnostic results comparable to those obtained with larger FOVs.

Given the increasing reliance on CBCT for periodontal purposes, it is critical to understand how combinations of voxel size and FOV (i.e., the acquisition protocols) impact the accuracy of alveolar bone measurements. To date, there is a lack of comparative studies that evaluate multiple CBCT systems using a standardized methodology for this application.

Therefore, this study aimed to investigate the influence of different CBCT acquisition protocols—specifically combinations of voxel size and FOV—on the accuracy of alveolar bone crest measurements in the context of periodontal diagnosis. This study employed four widely used CBCT systems and compared their performance using an ex vivo human mandible model under standardized conditions.

## 2. Materials and Methods

### 2.1. Study Design and Ethical Approval

This was an experimental, analytical, ex vivo study approved by the institutional ethics committee (protocol number: 6.198.104). A macerated human mandible specimen from the Department of Diagnosis and Surgery at São Paulo State University (UNESP) was used. CBCT scans were acquired at two private clinics and one university imaging department. A total of 64 CBCT volumes were obtained, distributed according to the availability of acquisition protocols in each tomographic system. Sample size calculation was based on a prior study [12], with a significance level of 5% and statistical power of 80%.

### 2.2. Specimen Preparation

Radiopaque reference points were created at the CEJ on the buccal–mesial and buccal–distal surfaces of teeth 34 and 43 using a mixture of zinc oxide powder (Preven^®^, Guapirama, Brazil), silver amalgam alloy (Luxalloy^®^, Degudent, Ciudad de Mexico, Mexico), and white glue (Tenaz^®^, Henkel, Dusseldorf, Germany), applied with a Dycal applicator [13]. These teeth were chosen based on their anatomical features and their reported susceptibility to advanced alveolar bone loss [14,15]. To simulate soft tissues, the mandible was covered with 2.0 cm of utility wax (Cera 7 Lysanda^®^, São Paulo, Brazil) on the buccal surface and 1.0 cm on the lingual side [16].

Linear measurements from the CEJ to the alveolar crest were performed directly on the specimen using a digital caliper (Mitutoyo, Kawasaki, Japan), establishing the gold standard. Each measurement was taken three times, and the mean values were used for comparison with those obtained from CBCT scans.

### 2.3. CBCT Image Acquisition

The mandible was positioned on a standardized rectangular base to maintain consistent orientation across all imaging systems. A scout view was obtained before each scan to ensure proper alignment of the region of interest at the center of the FOV. A custom A4 paper template with reference marks was used to replicate positioning across acquisitions.

Scans were performed on four CBCT systems:

- Veraview^®^ X800 (J. Morita MFG. Corp., Tokyo, Japan);
- OP300 Pro^®^ (Instrumentarium Dental, Tuusula, Finland);
- I-CAT Next Generation^®^ (Imaging Sciences International, Hatfield, PA, USA);
- Orthophos XG 5 (Dentsply Sirona, Bensheim, Germany).

**Figure 1:**
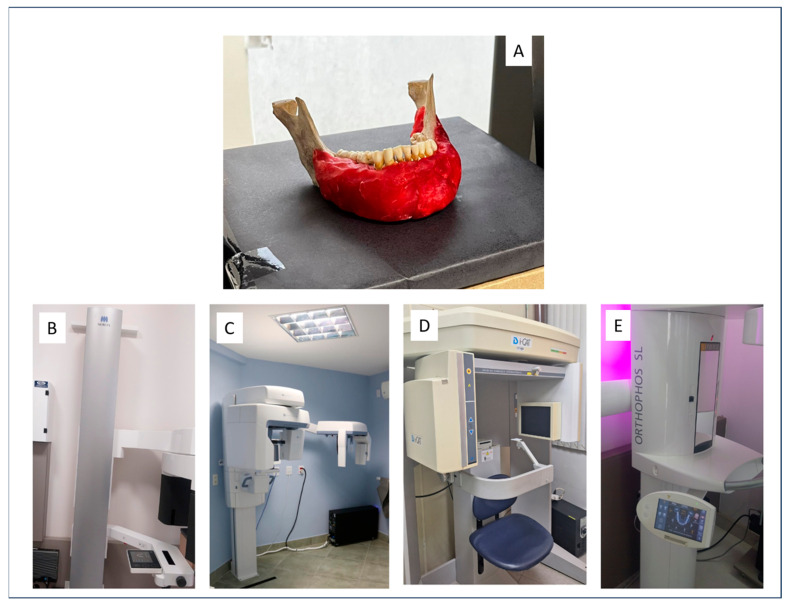
CBCT acquisition setup and systems used: (**A**) mandible positioned on the standardized rectangular base; (**B**) Veraview^®^ X800 (J. Morita MFG. Corp., Tokyo, Japan); (**C**) OP300 Pro^®^ (Instrumentarium Dental, Tuusula, Finland); (**D**) I-CAT Next Generation^®^ (Imaging Sciences International, Hatfield, PA, USA); (**E**) Orthophos XG 5 (Dentsply Sirona, Bensheim, Germany).

Figure 1 illustrates the experimental setup and CBCT systems used in this study. Panel A shows the standardized positioning of the mandible on a rectangular base to ensure consistent orientation across all image acquisitions. Panels B through E display the four CBCT systems evaluated: Veraview^®^ X800, OP300 Pro^®^, I-CAT Next Generation^®^, and Orthophos XG 5, respectively. The use of a customized positioning guide and pre-scan scout views ensured accurate and reproducible alignment of the region of interest in each system.

Protocol selection in each system was based on those most used in clinical settings and on achieving full coverage of a single mandibular arch. A total of 61 acquisition protocols combining varying FOVs and voxel sizes were tested (Table 1).

### 2.4. Image Processing and Measurements

All image analyses were conducted by a single trained examiner and calibrated for the evaluation protocol. Paracoronal slices were generated using OnDemand3D software (Cybermed, Tustin, CA, USA), with a 2× sharpening filter and standard window settings (WL = 1077, WW = 4083). Although CBCT provides three-dimensional imaging, this study was limited to two-dimensional linear measurements obtained from paracoronal slices. Although this method is widely used and offers high reproducibility, it does not fully leverage the volumetric capabilities of CBCT technology. Future research should explore the implementation of 3D volumetric segmentation and analysis techniques, which may enable a more comprehensive evaluation of periodontal bone morphology and spatial distribution.

In the software’s Dental module, each tooth was centered in the axial plane, and the slice orientation was adjusted to obtain optimal paracoronal views. Linear measurements were performed from the radiopaque CEJ marker to the alveolar crest using the built-in measurement tool. Each measurement was performed in triplicate and recorded in Microsoft Excel. Including the in situ measurements, a total of 642 data points were collected.

**Figure 2:**
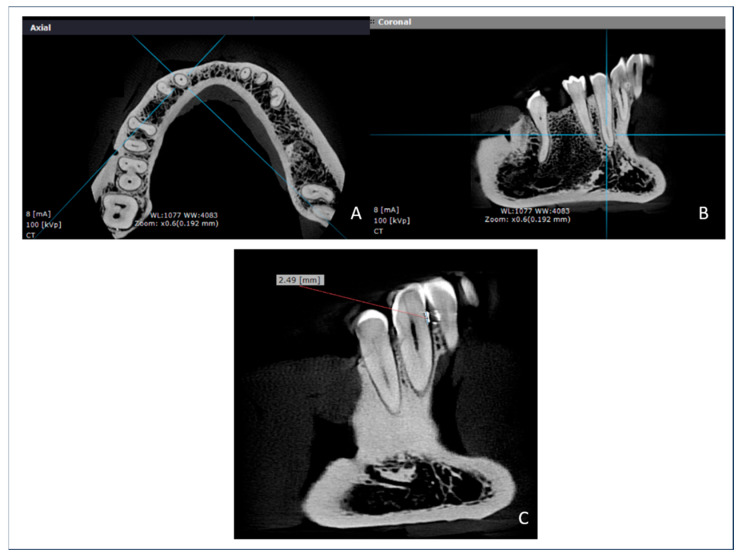
OnDemand3D software interface during image analysis: (**A**) axial reference slice used to adjust paracoronal orientation; (**B**) paracoronal slice of the region of interest prepared for measurement; (**C**) paracoronal slice with linear measurement from the CEJ marker to the alveolar crest.

Figure 2 presents representative screenshots from the OnDemand3D software, illustrating the image processing workflow used in this study. The axial view (Panel A) was used to define the orientation for paracoronal reformatting. Panel B shows a paracoronal slice of the region of interest, while Panel C demonstrates the linear measurement from the radiopaque CEJ marker to the alveolar crest. All measurements were performed in triplicate by a calibrated examiner using standardized window settings and image enhancement filters.

To assess intraobserver reliability, all measurements were repeated after a 30-day interval, yielding excellent intraclass correlation coefficient (ICC) values. All evaluations were performed in a controlled environment using a 27-inch LCD monitor under low-light conditions. The assessment of intraobserver reliability demonstrated excellent consistency, with an intraclass correlation coefficient (ICC) of 0.92 (95% confidence interval: 0.88–0.95). Beyond *p*-values, 95% confidence intervals (CIs) for the mean differences between each CBCT protocol and the gold standard were calculated to provide a more robust assessment of agreement and clinical relevance.

### 2.5. Statistical Analysis

Statistical analyses were performed using R software 3.6.0. (R Core Team, Vienna, Austria). Data normality was assessed using the Shapiro–Wilk test. Descriptive statistics included means, standard deviations, and minimum and maximum values. Inferential analysis was conducted using one-way ANOVA, followed by Dunnett’s test for comparisons with the gold standard. A significance level of *p* < 0.005 was adopted throughout.

## 3. Results

A total of 64 CBCT scans were analyzed across the four CBCT systems, using 61 different acquisition protocols. Linear measurements from the CEJ to the alveolar bone crest obtained from the CBCT images were compared to the in situ gold standard.

### 3.1. OP300 System

The OP300 system demonstrated a wide range of performance depending on protocol configuration (Table 2). Protocols with a smaller FOV and intermediate voxel size yielded the most accurate results. Specifically, the protocols with FOV = 6 × 8 cm and voxel = 0.2 mm (mean = 2.52 mm; *p* = 0.995) and FOV = 5 × 5 cm with voxel sizes of 0.125 mm and 0.085 mm (means = 2.56 mm for both; *p* = 1.000) showed the closest agreement with the gold standard (mean = 2.57 mm). In contrast, protocols with large FOVs and higher voxel values, such as FOV = 8 × 15 cm and voxel = 0.4 mm (mean = 3.13 mm; *p* < 0.001), significantly overestimated bone levels.

### 3.2. Veraview X800 System

For the Veraview X800 system (Table 3), most protocols yielded measurements not significantly different from the gold standard. The only exception was the protocol with FOV = 8 × 5 cm and voxel = 0.125 mm (mean = 2.24 mm; *p* = 0.019), which significantly underestimated bone height. Other small-FOV protocols, including FOV = 4 × 4 cm with voxel = 0.08 mm and FOV = 15 × 7.5 cm with voxel = 0.25 mm, produced results consistent with the gold standard.

### 3.3. Orthophos XG5 System

The Orthophos XG5 system showed homogeneous performance across all evaluated protocols (Table 4). No statistically significant differences were found between any of the CBCT protocols and the gold standard (ANOVA *p* = 0.092). All protocols, including FOVs of 5 × 5.5 cm, 8 × 8 cm, and 11 × 10 cm with voxel sizes of 0.08 mm and 0.16 mm, provided consistent measurements within acceptable limits of agreement.

### 3.4. I-CAT System

The I-CAT system displayed more variability in measurement accuracy (Table 5). Most protocols with large voxel sizes (e.g., voxel = 0.4 mm) overestimated bone levels, with means exceeding 3.0 mm and *p*-values < 0.001. However, the protocol using FOV = 7.1 × 16 cm and voxel = 0.25 mm (mean = 2.60 mm; *p* = 0.988), as well as the 0.2 mm voxel protocol (mean = 2.70 mm; *p* = 0.166), did not significantly differ from the gold standard, indicating adequate accuracy.

Table 6 presents the classification of all CBCT systems and acquisition protocols based on their agreement with the gold standard, providing a clearer interpretation of protocol accuracy.

## 4. Discussion

This study evaluated the influence of different CBCT acquisition protocols—specifically, combinations of voxel size and field of view (FOV)—on the accuracy of alveolar bone height measurements in a periodontal context. The results demonstrated that smaller voxel sizes and reduced FOVs generally improved measurement accuracy when compared to the in situ gold standard, but some intermediate-resolution protocols also yielded comparable performance, depending on the system used.

CBCT has become increasingly relevant in periodontal diagnostics, particularly for cases involving surgical planning, furcation defects, or alveolar dehiscence/fenestration [3,5,17,18,19]. However, the optimal CBCT parameters for such applications remain poorly defined. This study reinforces the need to tailor acquisition protocols to the diagnostic task, in alignment with the ALADA principle [9].

No data were collected or reported regarding the effective radiation dose associated with the various CBCT protocols evaluated. This limitation restricts the ability to fully assess the trade-off between diagnostic accuracy and radiation exposure. Incorporating even approximate dose estimates—whether derived from manufacturer specifications or prior literature—would strengthen the clinical applicability of the findings and support more informed protocol selection. Future research should include dosimetric analyses to compare effective radiation doses across CBCT devices and acquisition settings, enabling a more evidence-based application of the ALADA principle in periodontal imaging [9,20].

Among the systems evaluated, the Orthophos XG5 produced the most consistent measurements across all protocols, showing no significant differences compared to the gold standard. The Veraview X800 system showed high accuracy overall, although the protocol combining FOV = 8 × 5 cm and voxel = 0.125 mm significantly underestimated bone height, suggesting that image noise at small voxel sizes may degrade diagnostic performance [21,22].

The OP300 system revealed a more marked influence of acquisition settings on accuracy. Protocols with small FOVs (5 × 5 cm or 6 × 8 cm) and voxel sizes between 0.2 mm and 0.125 mm provided the best agreement with gold standard measurements. Conversely, protocols with small FOVs and voxel sizes (e.g., 8 × 15 cm, 0.4 mm voxel) significantly overestimated bone height, consistent with prior findings that large voxel sizes reduce spatial resolution and compromise diagnostic utility [23,24,25].

The I-CAT system exhibited the lowest agreement overall, especially in protocols with higher voxel values. However, protocols using voxel sizes of 0.2 mm and 0.25 mm demonstrated acceptable performance, suggesting that this system, while less stable, can yield reliable measurements under optimized conditions.

These findings align with previous studies reporting that smaller voxel sizes—typically below 0.2 mm—are associated with improved detection of fine anatomical details, including root resorptions and periodontal defects [24,25,26,27]. Nonetheless, evidence also suggests that extremely small voxel sizes may introduce higher noise levels or demand increased radiation exposure, without proportionate gains in diagnostic accuracy [20,22,26].

Notably, our results highlight that diagnostic reliability does not necessarily linearly increase with image resolution. Some intermediate protocols achieved comparable accuracy to high-resolution scans, as similarly reported by Liedke et al. [24], reinforcing the notion that “low-dose” CBCT settings may be sufficient for specific periodontal applications [25,27,28,29].

In dental practice, it is essential to maintain a balance between diagnostic precision, accurate measurement of anatomical structures, and minimizing patient radiation exposure, in accordance with the ALADA principle [9]. When the clinical objective involves detailed evaluation of root canal anatomy—for example, in endodontic treatment planning—the use of smaller voxel sizes is justified, despite the associated increase in radiation dose [30]. Conversely, in the assessment of expansive lesions affecting the maxillofacial complex, such as ameloblastomas, the use of protocols with reduced FOV and voxel size can be employed with lower radiation exposure, without compromising diagnostic interpretation [31].

A key strength of this study was the inclusion of four widely used CBCT systems and 61 acquisition protocols, enabling comprehensive inter-system comparison under standardized conditions. The use of radiopaque CEJ markers and simulated soft tissues allowed for reproducible and clinically relevant measurement scenarios.

One limitation of this study is its ex vivo design. Although the use of a dry mandible minimized motion artifacts and ensured protocol standardization, it does not replicate intraoral clinical conditions, where patient movement and anatomical variability may affect image quality. Furthermore, due to technical constraints, we were unable to evaluate effective radiation dose or compare amperage and kilovoltage across all devices—factors that also influence diagnostic performance.

Although a standardized rectangular base, scout views, and a paper template were used to ensure consistent mandible positioning across CBCT systems, no formal digital image registration or software-based alignment was performed. This setup allowed for general alignment of the region of interest but did not achieve precise spatial registration between scans. The lack of 3D image registration may have introduced variability in slice orientation and measurement positioning, potentially affecting the comparability of linear measurements across devices. We acknowledge this as a methodological limitation. Prior studies have shown that even minor deviations in head or object positioning can influence CBCT measurements and diagnostic interpretation [19,29]. Future research should consider the use of fiducial markers or software-based registration tools (e.g., DICOM-to-DICOM alignment) to improve positional consistency and enhance measurement reliability in multi-device comparisons [26].

Additionally, in vivo CBCT scans are subject to patient motion, which can generate artifacts and degrade image quality—especially when using small voxel sizes or extended exposure times [8,29]. The absence of heterogeneous soft tissues and metallic restorations in our experimental setup may also have contributed to an idealized imaging scenario. In clinical conditions, soft tissue variability and the presence of metallic objects (e.g., amalgam fillings or implants) may induce scattering and beam hardening artifacts, which can obscure anatomical details and reduce diagnostic accuracy [5,9,26]. Therefore, while the present findings provide valuable insights under controlled conditions, further validation in vivo is essential to confirm their applicability in clinical practice.

## 5. Conclusions

Protocols combining smaller voxel sizes with limited FOVs provided the most accurate measurements of alveolar bone levels in this study. A voxel size of 0.125–0.2 mm and reduced FOVs appear to offer a favorable balance between diagnostic accuracy and radiation exposure. Among the four CBCT systems tested, Orthophos XG5 and Veraview X800 showed more consistent agreement with the gold standard. CBCT should be considered a reliable imaging modality for periodontal bone assessment when appropriately optimized.

## Figures and Tables

**Table 1:** Acquisition protocols and technical specifications used in the OP300, Veraview X800, Orthophos XG5, and I-CAT Next Generation CBCT systems included in this study. FOV = field of view (H = height, D = diameter), both in centimeters; voxel size measured in millimeters; SAR = standard acquisition mode (without artifact reduction); AR = acquisition with artifact reduction; RS = right side; LS = left side; kVp = kilovoltage peak; mA = milliamperage; exposure time given in seconds.

CBCT System	FOV (H × D) (cm)	Voxel (mm)	Specifications (kVp/mA/s)
OP300	8 × 15	0.4^SAR/^0.32^SAR/^0.25^SAR^/0.4^AR^ 0.32^AR^/0.25^AR^	90 kVp/3.2 mA/2.3 s 90 kVp/6.3 mA/4.5 s
	8 × 8	0.32^SAR^/0.3^SAR^/0.2^SAR^/0.32^AR^ 0.3 ^AR^/0.2 ^AR^	90 kVp/3.2 mA/1.2 s 90 kVp/8.0 mA/2.3 s
	6 × 8	0.32^SAR^/0.3^SAR^/0.2^SAR^/0.32^AR^ 0.3 ^AR^/0.2 ^AR^	90 kVp/3.2 mA/1.2 s 90 kVp/8.0 mA/2.3 s
	5 × 5	0.28 ^SAR. AR. LE^/0.2 ^SAR. AR. LE^/0.125 ^SAR. AR. LE^ 0.085 ^SAR. AR. LE^/0.28 ^SAR. AR. LD^/0.2 ^SAR. AR. LD^ 0.125 ^SAR. AR. LD^/0.085 ^SAR. AR. LD^	90 kVp/3.2 mA/1.2 s 90 kVp/6.3 mA/8.7 s 90 kVp/6.3 mA/6.1 s
	13 × 15	0.42 ^AR^/0.38 ^AR^/0.32 ^AR^/0.48^SAR^ 0.38^SAR^/0.32^SAR^	90 kVp/3.2 mA/4.5 s 90 kVp/5.0 mA/8.1 s
Veraview X800	4 × 4 (Endo)	0.08 ^RS. LS^	100 kVp/8.0 mA/10.0 s
	4 × 4	0.08 ^RS. LS^	100 kVp/8.0 mA/8.5 s
	8 × 4/8 × 5/8 × 8/ 10 × 4/10 × 5/10 × 8	0.125	100 kVp/8.0 mA/8.3 s 100 kVp/8.0 mA/7.6 s
	15 × 5/15 × 7.5	0.25	101 kVp/8.0 mA/2.5 s
Ortophos XG5	5 × 5.5	0.08 ^RS. LS^	85 kVp/7.0 mA/7.5 s
	8 × 8/11 x10	0.16	85 kVp/6.0 mA/5.8 s
I-CAT Next Generation	7.1 × 16	0.4/0.3/0.25/0.2	120 kVp/36 mA/8.0 s
	6.1 × 16	0.4	89 kVp/8.0 mA/12.0 s

**Table 2:** Summary comparison of OP300 CBCT protocols with the gold standard (teeth 34 and 43).

Protocol	Mean	Standard Deviation	Minimum	Maximum	Protocol
FOV = 6 × 8 Res = 0.3	2.50	0.36	1.73	3.05	0.940
FOV = 6 × 8 Res = 0.2	2.52	0.13	2.30	2.74	0.995
FOV = 5 × 5 Res = 0.125	2.56	0.13	2.37	2.75	1.000
FOV = 5 × 5 Res = 0.085	2.56	0.11	2.38	2.77	1.000
FOV = 5 × 5 Res = 0.2	2.65	0.14	2.37	2.91	0.911
FOV = 8 × 8 Res = 0.2	2.65	0.19	2.33	2.95	0.862
FOV = 8 × 15 Res = 0.25	2.69	0.14	2.38	2.88	0.526
FOV = 8 × 8 Res = 0.32	2.73	0.20	2.40	3.20	0.190
FOV = 5 × 5 Res = 0.28	2.76	0.18	2.35	3.03	0.071
FOV = 8 × 8 Res = 0.3	2.76	0.23	2.41	3.19	0.081
FOV = 13 × 15 Res = 0.32	2.82	0.20	2.50	3.30	**0.007**
FOV = 6 × 8 Res = 0.32	2.84	0.19	2.50	3.20	**0.003**
FOV = 13 × 15 Res = 0.38	2.85	0.22	2.50	3.40	**0.001**
FOV = 8 × 15 Res = 0.32	2.95	0.21	2.40	3.20	**<0.001**
FOV = 13 × 15 Res = 0.42	2.97	0.28	2.50	3.40	**<0.001**
FOV = 8 × 15 Res = 0.4	3.13	0.22	2.80	3.60	**<0.001**
**Gold standard**	**2.57**	**0.20**	**2.30**	**2.97**	

FOV = field of view; Res = spatial resolution. Units: voxel (mm) and FOV (cm). Bold values are significant at *p* < 0.005.

**Table 3:** Summary comparison of Veraview X800 CBCT protocols with the gold standard (teeth 34 and 43).

Protocol	Mean	Standard Deviation	Minimum	Maximum	Protocol
FOV = 8 × 5 Res = 0.125	2.24	0.26	1.93	2.60	**0.019**
FOV = 8 × 8 Res = 0.125	2.32	0.35	1.73	2.88	0.133
FOV = 8 × 4 Res = 0.125	2.34	0.30	1.97	2.86	0.200
FOV = 10 × 5 Res = 0.125	2.38	0.18	2.11	2.69	0.387
FOV = 15 × 5 Res = 0.25	2.38	0.16	2.17	2.69	0.392
FOV = 10 × 8 Res = 0.125	2.39	0.31	1.91	2.83	0.438
FOV = 10 × 4 Res = 0.125	2.50	0.31	1.97	2.86	0.989
FOV = 4 × 4 Res = 0.08	2.50	0.30	1.97	2.93	0.965
FOV = 15 × 7.5 Res = 0.25	2.54	0.17	2.31	2.89	1.000
**Gold standard**	**2.57**	**0.20**	**2.30**	**2.97**	

FOV = field of view; Res = spatial resolution. Units: voxel (mm) and FOV (cm). Bold values are significant at *p* < 0.005.

**Table 4:** Summary comparison of Orthophos XG5 CBCT protocols with the gold standard (teeth 34 and 43).

Protocol	Mean	Standard Deviation	Minimum	Maximum
FOV = 5 × 5.5 Res = 0.08	2.35	0.26	1.93	2.70
FOV = 11 × 10 Res = 0.16	2.39	0.10	2.25	2.58
FOV = 8 × 8 Res = 0.16	2.41	0.29	1.93	2.72
**Gold standard**	**2.57**	**0.20**	**2.30**	**2.97**

FOV = field of view; Res = spatial resolution. Units: voxel (mm) and FOV (cm). Bold values are significant at *p* < 0.005.

**Table 5:** Summary comparison of I-CAT CBCT protocols with the gold standard (teeth 34 and 43).

Protocol	Mean	Standard Deviation	Minimum	Maximum	*p*-Value
FOV = 7.1 × 16 Res = 0.4	3.22	0.35	2.80	3.70	**<0.001**
FOV = 6.1 × 16 Res = 0.4	3.00	0.12	2.80	3.20	**<0.001**
FOV = 7.1 × 16 Res = 0.3	2.74	0.15	2.51	3.08	**0.039**
FOV = 7.1 × 16 Res = 0.2	2.70	0.10	2.50	2.88	0.166
FOV = 7.1 × 16 Res = 0.25	2.60	0.18	2.08	2.89	0.988
**Gold standard**	**2.57**	**0.20**	**2.30**	**2.97**	

FOV = field of view; Res = spatial resolution. Units: voxel (mm) and FOV (cm). Bold values are significant at *p* < 0.005.

**Table 6:** Summary classification of all CBCT acquisition protocols used in this study, categorized by acquisition mode (SAR, AR) and scan orientation (right and left sides), based on their statistical agreement with the gold standard (mean = 2.57 mm). Classification criteria: accurate (*p* ≥ 0.05); moderately different (0.01 ≤ *p* < 0.05); significantly different (*p* < 0.01).

CBCT System	Protocol (FOV/Voxel)	Mode/Side	Mean (mm)	*p*-Value	Classification
OP300	5 × 5/0.125	SAR, AR, LE	2.56	1.000	Accurate (+ +)
	5 × 5/0.125	SAR, AR, LD	2.56	1.000	Accurate (+ +)
	5 × 5/0.085	SAR, AR, LE	2.56	1.000	Accurate (+ +)
	5 × 5/0.085	SAR, AR, LD	2.56	1.000	Accurate (+ +)
	6 × 8/0.2	SAR	2.52	0.995	Accurate (+ +)
	13 × 15/0.32	AR	2.82	0.007	Moderately different (+ −)
	13 × 15/0.38	AR	2.85	0.001	Significantly different (− −)
	8 × 15/0.4	SAR	3.13	<0.001	Significantly different (− −)
Veraview X800	4 × 4/0.08	RS, LS	2.50	0.965	Accurate (+ +)
	15 × 7.5/0.25	Standard	2.54	1.000	Accurate (+ +)
	8 × 5/0.125	Standard	2.24	0.019	Moderately different (+ −)
Orthophos XG5	5 × 5.5/0.08	RS, LS	2.35	>0.05	Accurate (+ +)
	8 × 8/0.16	Standard	2.41	>0.05	Accurate (+ +)
	11 × 10/0.16	Standard	2.39	>0.05	Accurate (+ +)
I-CAT Next Generation	7.1 × 16/0.25	Standard	2.60	0.988	Accurate (+ +)
	7.1 × 16/0.2	Standard	2.70	0.166	Accurate (+ +)
	7.1 × 16/0.3	Standard	2.74	0.039	Moderately different (+ −)
	7.1 × 16/0.4	Standard	3.22	<0.001	Significantly different (− −)
	6.1 × 16/0.4	Standard	3.00	<0.001	Significantly different (− −)

The symbols “+” and “−” in the classification column indicate the direction of agreement or deviation from the gold standard (mean = 2.57 mm). “+” denotes that the mean measurement was within or below the gold standard range, while “−” indicates an overestimation. Double signs (e.g., “+ +” or “− −”) represent consistency across both acquisition sides or modes; mixed signs (e.g., “+ −”) reflect variation between left and right sides or modes.

## Data Availability

The datasets generated during and/or analyzed during the current study are available from the corresponding author upon reasonable request.
